# Supplementary figures and images for: A laboratory-adapted and a clinical isolate of dengue virus serotype 4 differently impact Aedes aegypti life-history traits relevant to vectorial capacity
Source: Microbiol Spectr. 2025 Oct 28;13(12):e00001-25. doi: 10.1128/spectrum.00001-25 (PMC12671126; doi:10.1128/spectrum.00001-25)

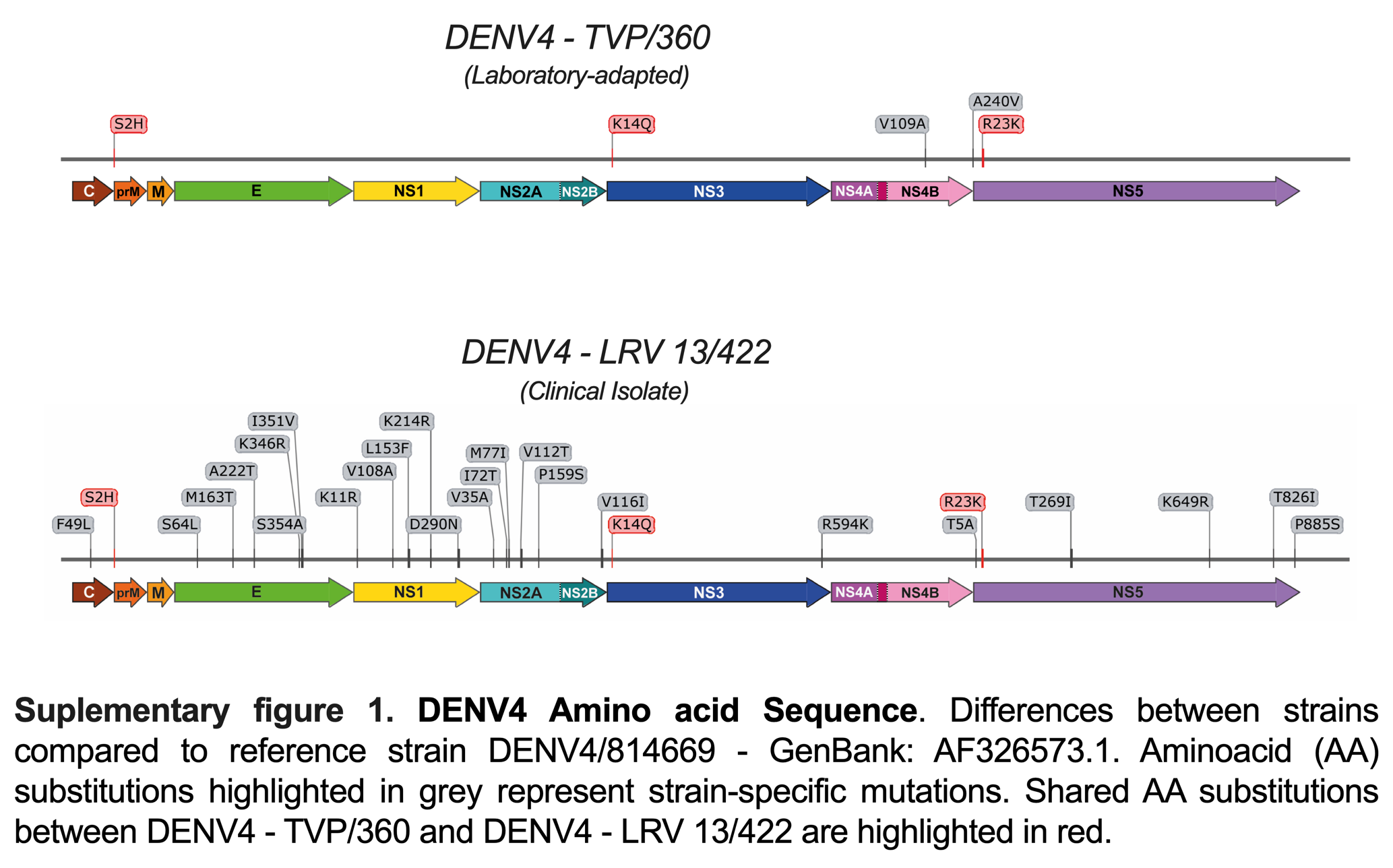

Supplement: Figure S1 — DENV4 amino acid sequence. [file spectrum.00001-25-s0001.tiff]

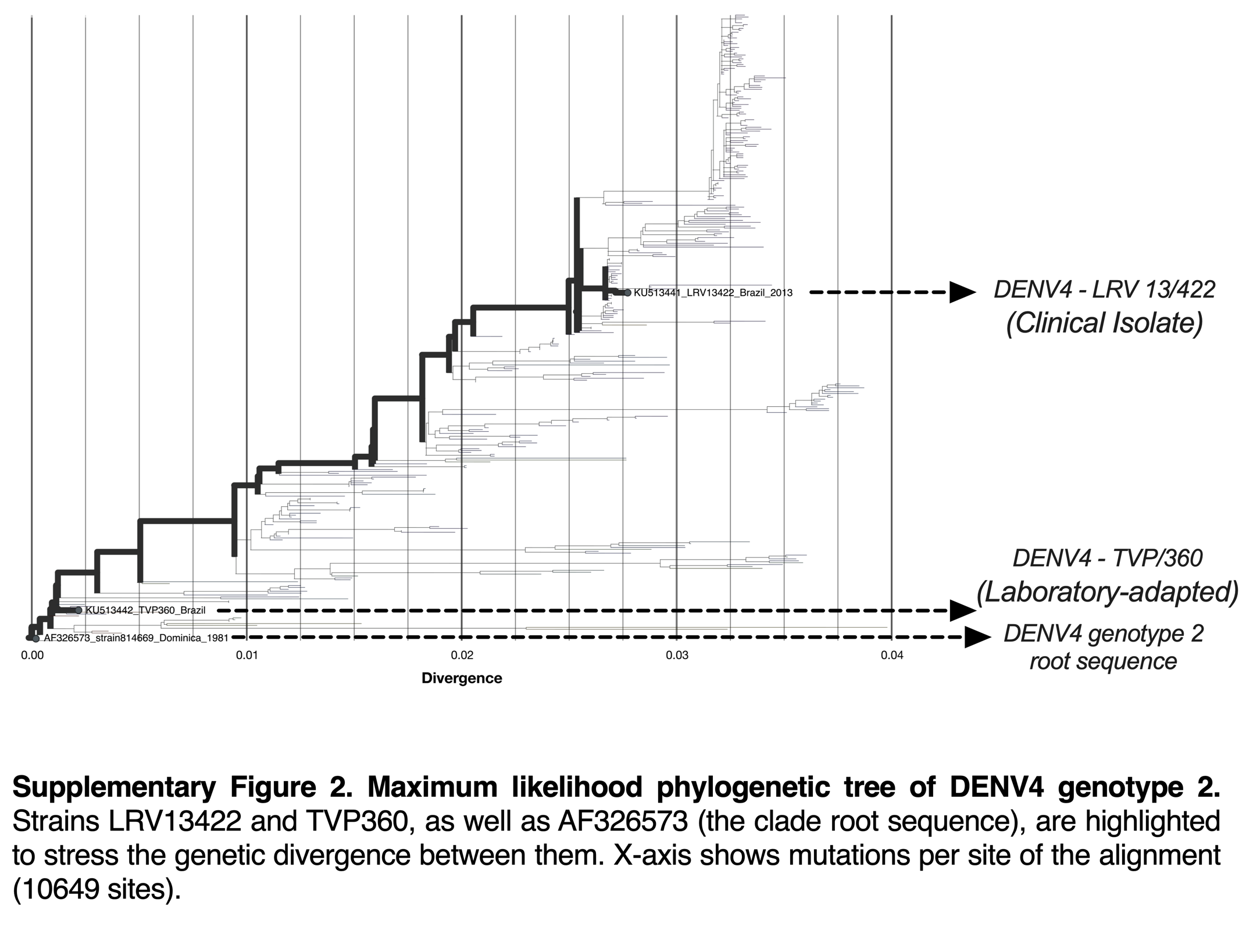

Supplement: Figure S2 — Maximum likelihood phylogenetic tree of DENV4 genotype 2. [file spectrum.00001-25-s0002.tiff]

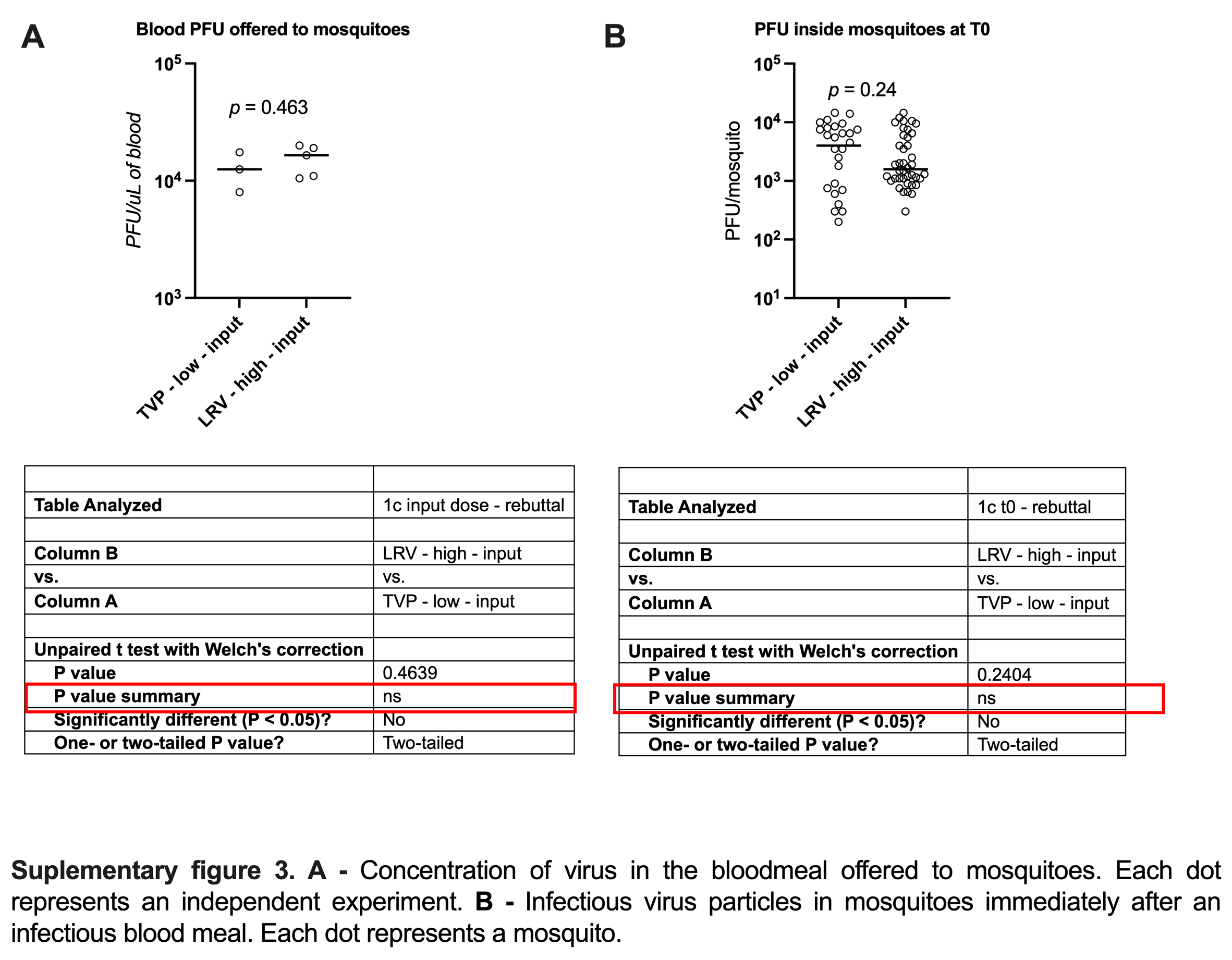

Supplement: Figure S3 — DENV4 viral loads offered to mosquitos and inside mosquitos immediately after feeding. [file spectrum.00001-25-s0003.tiff]

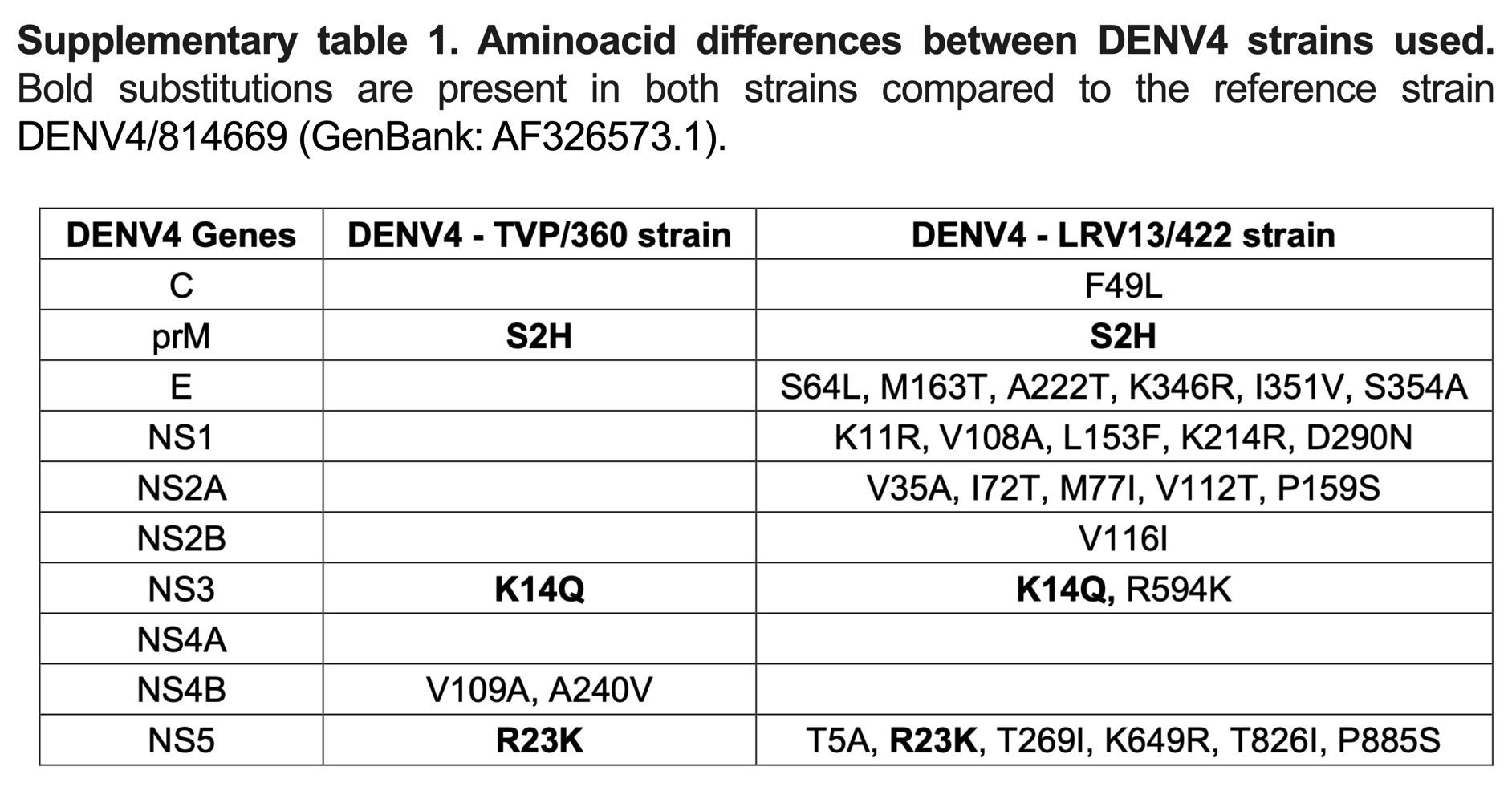

Supplement: Table S1 — Amino acid differences between DENV4 strains used. [file spectrum.00001-25-s0004.tiff]
